# HSP40 Interacts with Pyruvate Kinase M2 and Regulates Glycolysis and Cell Proliferation in Tumor Cells

**DOI:** 10.1371/journal.pone.0092949

**Published:** 2014-03-21

**Authors:** Liangqian Huang, Zhenhai Yu, Teng Zhang, Xiaoping Zhao, Gang Huang

**Affiliations:** 1 Institute of Health Sciences, Shanghai Institutes for Biological Sciences (SIBS), Chinese Academy of Sciences (CAS) & Shanghai Jiao Tong University School of Medicine (SJTUSM), Shanghai, China; 2 Department of Nuclear Medicine, Renji Hospital, School of Medicine, Shanghai Jiao Tong University, Shanghai, China; 3 School of Biomedical Engineering, Shanghai Jiao Tong University, Shanghai, China; Southern Illinois University School of Medicine, United States of America

## Abstract

Pyruvate kinase M2 (PKM2) is predominantly expressed in cancers, which is considered as a key regulator of the Warburg effect. In this study, HSP40 was identified as a novel binding partner of PKM2. HSP40-PKM2 association destabilized PKM2 protein through HSC70. In the presence of HSP40, PKM2 protein level and PKM2-mediated PDK1 expression were down-regulated. Moreover, HSP40 was involved in regulating glucose metabolism on PKM2 dependent way and at the mean time had an effect on mitochondrial oxygen respiration. In line with inhibition effect of HSP40 on glycolysis, the growth of cancer cells was inhibited by HSP40.Our data provided a new regulation mechanism of PKM2, which suggested a new therapeutic target for cancer therapy.

## Introduction

Altered energy metabolism is proved to be widespread in cancer cells that have been accepted as an emerging hallmark of cancer [1]. As first observed by Otto Warburg, cancer cells have elevated rates of glucose consumption and high lactate production in the presence of oxygen, known as aerobic glycolysis (Warburg Effect) [2]. High glucose uptake is used clinically to diagnose and monitor treatment responses of cancers by imaging uptake of 2-^18^F-deoxyglucose with PET [3]. Despite its wide clinical applications, the mechanisms underlying the Warburg effect remain largely elusive. Mutations of key components of several signaling pathways and metabolic enzymes have been thought to play significant roles in cancer metabolic reprogramming [4], [5].

Pyruvate kinase (PK) is a key rate-limiting enzyme of glycolysis which catalyzes the final step of glycolysis. It converts phosphoenolpyruvate (PEP) to pyruvate while phosphorylating ADP to ATP. There are four isoforms of pyruvate kinase, including PKL, PLR, PKM1 and PKM2 [6]. During tumorigenesis, tissue-specific PKM1/L/R expression gradually diminishes and is replaced by PKM2 expression. PKM2 is highly expressed in nearly all cancer cells [7]. It is considered as a key regulator of Warburg effect. PKM2 also functions as a transcriptional co-activator or a protein kinase to regulate tumorigenesis [8]–[10]. However, the molecular mechanisms underlying the regulation of PKM2 need to be further clarified.

Heat shock proteins (HSPs) are up-regulated while cells are exposed to elevated temperatures or oxygen deprivation [11]. The members of this family have been conserved throughout evolution. They are indispensable for protein translation, folding, unfolding, translocation, and degradation [12]. They are involved in the cell metabolism, cell cycle and others [13]. Previous studies show that HSP members are implicated in tumorigenesis, including tumor suppressors HLJ1 (DNAJB4), Tid1 (DNAJA3), DNAJC25, radio-resistance factor HDJ2 (DNAJA1) and other tumor-related members such as DNAJB6, DNAJC12, DNAJC1, DNAJC12, DNAJC15 [14]–[17]. The studies of HSPs diagnosis and treatment in cancer suggest that they are novel therapeutic targets [18], [19].

Considering the critical role of PKM2 in tumorigenesis, this study was directed toward to understand the underlying mechanisms of regulation of PKM2. In our study, HSP40 was found to be associated with PKM2 via yeast two-hybrid screening. Our results indicated that HSP40-PKM2 association was related to pyruvate kinase activity and PKM2-mediated glycolytic gene expression. Our findings provided new insight into mechanism underlying regulation of PKM2 by HSP40, which correlated with receding cancer cell growth through glucose metabolic reprogramming.

## Materials and Methods

### Yeast Two-Hybrid Screening

The full-length, one N-terminal (1–354aa) and two C-terminal portions (354–531aa, 406–531aa) of human PKM2 were cloned into yeast expression vector pGBKT7 (Clontech). These constructs were used as baits for the screening of Human Kidney cDNA Library (Catalog No. 638816, Clontech). The screening for the interacting protein candidates by yeast two-hybrid was performed according to the manufacturer's instructions (Clontech).

### Cell Culture and Transfection

All cell lines including HEK293T, HeLa, A549, HepG2 were cultured in DMEM (GIBCO) supplemented with 10% FBS (GIBCO) at 37 °C in a humidified atmosphere of 5% CO_2_. Hypoxic treatment was performed in a specially designed hypoxia incubator (Thermo Electron) with 1.5% O_2_, 5% CO_2_ and 93.5% N_2_. Transfection of plasmids or siRNAs was performed by Lipofectamine 2000 (Invitrogen) following the manufacture's instruction. Full-length PKM2 and HSC70 were cloned into pCDNA3.0-HA vector and full-length HSP40/DNAJB1 was cloned into pFlag-CMV-4 vector. PKM2 siRNA and HSP40 siRNAs were purchased from Genepharma. PKM2 siRNA was generated with CATCTACCACTTGCAATTA oligonucleotide targeting exon 10 of the PKM2 transcript [9]. Three HSP40 siRNAs were used in our work, one HSP40 siRNA was designed by Genphama (1#) and the others were synthesized based on the published works (2# and 3#) [20], [21]:

1#: 5′-CAGCUGAUAUCGUCUUUCUTT-3′;

2#: 5′-CGACGGAAAGAGCATTCGATT-3′;

3#: 5′-ACCCGUCGUAUUCAAAGAUGUTT-3′.

### Immunoprecipitation, Immunoblotting, Immunofluorescence and Antibodies

For co-immunoprecipitation experiments, HEK293T or HeLa cells were lysed by IP cell lysis buffer (P0013, Beyotime) containing certain protease inhibitors. The whole cell lysate were incubated with indicated antibodies together with 20 ul protein A plus-agarose (#20333, Pierce) overnight at 4 °C. Immunoprecipitates were washed three times and resuspended in 20 ul of 2X SDS loading buffer, then resolved by SDS-PAGE after heated at 100 °C for 10 min [22]. For immunofluorescence assay, HeLa cells were transfected with indicated plasmids, and after 24 h incubation, cells were harvested for immunofluorescence indicated by manufacturer's instructions (Abcam). The localization of interesting proteins was detected by confocal laser scanning microscope (Leica TCS SP5). Antibodies and IgG used in this study were purchased as follows: anti-HSP40 (#13174-1-AP, Proteintech), anti-PKM2 (Abcam), anti-HA (#M20003, Abmart), anti-Flag (#M20008, Abmart and GTX115043, GeneTex), anti-β-actin (#P30002, Abmart), Goat anti-Mouse second antibody IRDye 800CW (#926-32220, LI-COR) and Goat anti-Rabbit second antibody IRDye 680RD (#926-32211, LI-COR), normal Mouse IgG (sc-2025, Santa Cruz).

### RNA Extraction and Semi-Quantitative RT-PCR

Total RNA was isolated by TRIZOL kit (Omega), and cDNA was synthesized by the cDNA synthesis kit (Takara). Quantitative real-time PCR was performed using the SYBR Green PCR Master Mix (Takara) on the Roche 480 system (Roche). Data were normalized to expression of a control gene (β -actin) for each experiment. Data represent the mean±SD of three independent experiments.

The sequences of the forward and reverse primers were as follows: PKM2 F, 5′-GTCGAAGCCCCATAGTGAAG-3′; R, 5′-GTGAATCAATGTCCAGGCGG-3′,

PDK1F, 5′-ACCAGGACAGCCAATACAAG-3′; R, 5′- CCTCGGTCACTCATCTTCAC-3′;

β-actin F, 5′-ACCAGGACAGCCAATACAAG-3′,R, 5′-CCTCGGTCACTCATCTTCAC- 3′.

### Glucose Uptake and Lactate Production Measurements

HeLa, A549 and HepG2 cells were seeded in 6-well plates and transfected with plasmids or siRNAs as indicated for 48 h, and followed with incubation in DMEM without FBS for 12 h. Medium was collected to detect glucose uptake and lactate production. Glucose uptake was measured using the glucose assay kit (Sigma) and lactate production was measured using the lactate assay kit (CMA, Microdialysis). Data were normalized by BCA protein measurements (P0010, Beyotime) at the end of experiments.

### Cellular Oxygen Consumption Rate Measurements

Oxygen consumption rate measurements were performed using the Seahorse XF24 Extracellular Flux Analyzer to monitor mitochondria respiration in real time. HeLa, A549 and HepG2 cells were plated in XF24 cell culture plates (Seahorse Bioscience) at 2.0×10^4^ cells/well and incubated for 24 h in a normal incubator. Then cells were equilibrated with bicarbonate-free low buffered DMEM medium in a 37 °C non CO_2_ incubator for 60 min immediately before XF assay. Perturbation compounds were prepared in the identical assay medium and were injected from the reagent ports automatically to the wells at the time as indicated [23].

### Statistical Analysis

The experiments were done at least three times. And error bars were represented mean±SD for triplicate experiments. Comparisons between groups were analyzed by unpaired t-tests (Student's t-tests) by Graphpad Prism 5. P value less than 0.05 was considered significant.

## Results

### HSP40 was identified as a novel PKM2 binding partner

**Figure 1:**
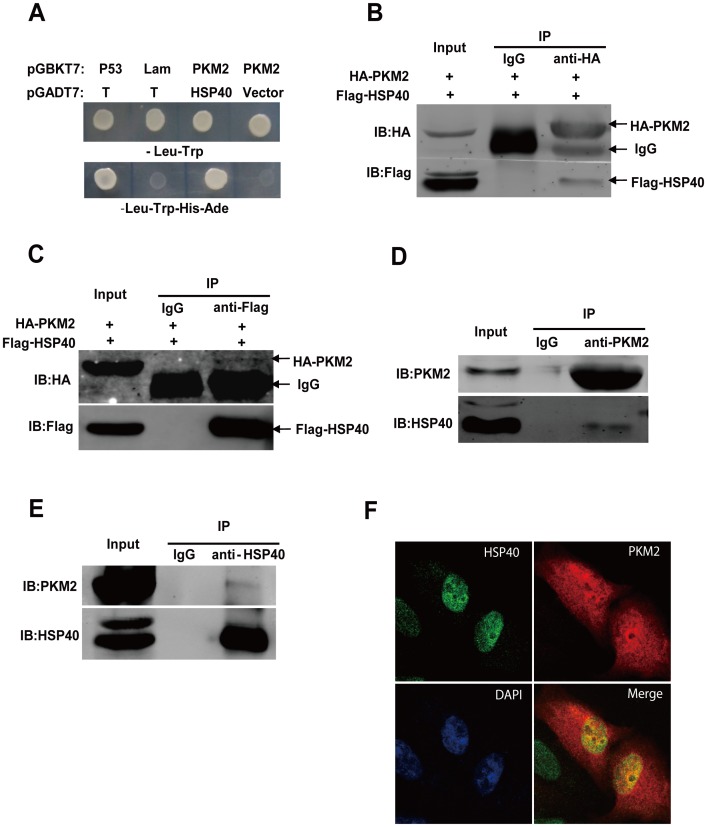
PKM2 interacts with HSP40/DNAJB1 in vivo and in vitro. (A) Growth of yeast expressing HSP40/DNJB1-GalAD and PKM2-GalBD on nutritional deficient SD medium (-Trp-Leu or -Trp-Leu-Ade-His). Vector pGADT7-T and pGBKT7-p53 performed as positive control, pGADT7-T and pGBKT7-Lam performed as negative control. (B) Cell lysates of HEK293T cells transfected with HA-PKM2 and Flag-HSP40 were immunoprecipitated with anti-HA antibody, the bound proteins were detected by anti-Flag antibodies. (C) Cell lysates of HEK293T cells transfected with HA-PKM2 and Flag-HSP40 were immunoprecipitated with anti-Flag antibody, the bound proteins were detected by anti-HA antibodies. (D) HeLa cells were lysed and Co-IP was performed with anti-PKM2 antibody, then western blot was analyzed using anti-PKM2 and anti-HSP40 antibodies. (E) HeLa cells were lysed and Co-IP was performed with anti-HSP40 antibody, then western blot was analyzed using anti-PKM2 and anti-HSP40 antibodies. (F) Immunofluorescence analysis reveals that HSP40 and PKM2 co-localize with each other in nucleus in HeLa cells.

To identify PKM2 interacting proteins, yeast two-hybrid was applied for screening. The PKM2 full length, one N-terminal fragment (1-354aa) and two C-terminal fragments (354-531aa and 406-531aa) were used as baits to screen a human kidney cDNA library [24], [25]. Two C-terminal fragments of PKM2 baited many prey proteins from the library. We extracted and sequenced the positive clones and listed in Table 1. Among all these candidates, HSP40/DNAJB1, a molecular chaperone of 40-kd heat shock protein (HSP40) was identified as a novel PKM2 binding partner. Both HSP40 prey vector and PKM2 bait vector were required for yeast growth in nutritional deficient SD medium (Fig. 1A).

**Table 1:** Genetic interactors of C-term PKM2 identified by yeast two-hybrid screening.

Abbreviation	Gene Name	Accession	No. of Gene Detected
EPC1	Homo sapiens enhancer of polycomb homolog 1	NM_001272004.1	1
MAN2B1	Homo sapiens mannosidase, alpha, class 2B, member 1	NM_001173498.1	1
C19orf60	Homo sapiens chromosome 19 open reading frame 60	NM_001100418.1	2
RASSF4	Homo sapiens Ras association (RalGDS/AF-6) domain family member 4	NM_032023.3	1
HTRA2	Homo sapiens HtrA serine peptidase 2	NM_013247.4	1
DHRS12	Homo sapiens dehydrogenase/reductase (SDR family) member 12	NM_001270424.1	1
POMP	Homo sapiens proteasome maturation protein	NM_015932.5	1
EBI3	Homo sapiens Epstein-Barr virus induced 3	NM_005755.2	1
CTSZ	Homo sapiens cathepsin Z	NM_001336.3	1
DOM3Z	Homo sapiens dom-3 homolog Z	NM_005510.3	1
C11orf1	Homo sapiens chromosome 11 open reading frame 1	NM_022761.2	1
Hsp40	Homo sapiens DnaJ (Hsp40) homolog, subfamily B, member 1	NM_006145.1	2
KANSL2	Homo sapiens KAT8 regulatory NSL complex subunit 2	NM_017822.3	1

To determine whether PKM2 interacted with HSP40 in mammalian cells, HA-PKM2 and Flag-HSP40 were co-transfected into HEK293T cells. The co-immunoprecipitation experiment revealed that their interaction also occurred in mammalian cells (Fig. 1B and 1C). More importantly, the binding of PKM2-HSP40 was also confirmed by endogenous co-immunoprecipitation assay in HeLa cells (Fig. 1D and 1E). To further reveal the details of PKM2-HSP40 interaction in cancer cells, confocal microscopy was used to detect their subcellular localization. PKM2 protein was dispersed in whole cell, which was consistent with its roles in both cytoplasm and nucleus [26]. Meanwhile, HSP40 predominantly localized in nucleus. As shown in Fig. 1F, co-localization between PKM2 and HSP40 was found mainly in nucleus. These findings indicated that PKM2 bound to the molecular chaperone HSP40.

### PKM2 protein level and functions were regulated by HSP40

**Figure 2:**
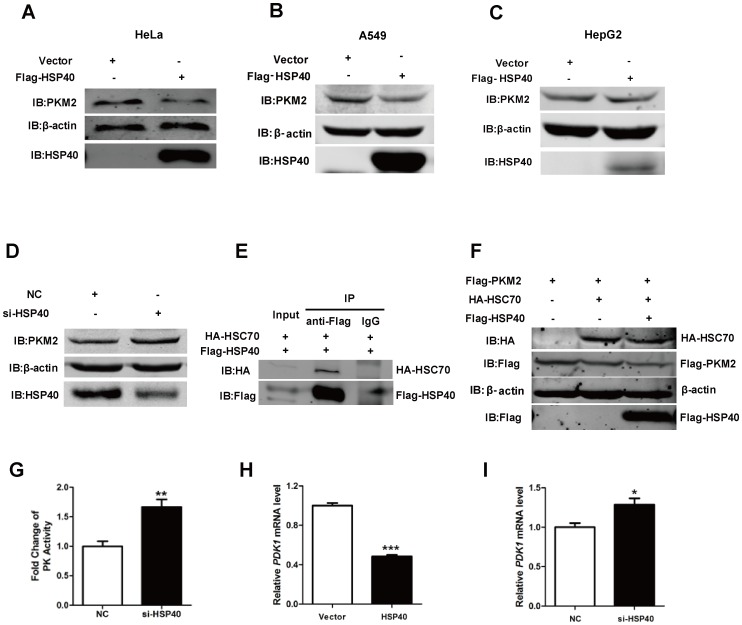
HSP40 impairs PKM2 stability and functions in HeLa cells. (A, B and C) HeLa, A549 and HepG2 cells were transfected with Flag-HSP40, after 48 h incubation, endogenous PKM2 protein levesl were detected by anti-PKM2 antibody. (D) HeLa cells were transfected with HSP40 siRNA, after 48 h incubation, PKM2 protein level was analyzed using anti-PKM2 antibody. (E) Cell lysates of HEK293T cells transfected with HA-HSC70 and Flag-HSP40/DNAJB1 were immunoprecipitated with anti-Flag antibody, the bound proteins were detected by anti-HA antibody. (F) HeLa cells transfected with HA-HSC70 or both HA-HSC70 and Flag-HSP40 were lysed, then western blot was analyzed using anti-PKM2, anti-HA and anti-Flag antibodies. (G) Pyruvate kinase activity of extracts prepared from HeLa cells transfected with HSP40 siRNA. The pyruvate kinase activity is detected by PK assay kit and normalized by protein measurement (mean ± S.D., n = 3). (H and I) HeLa cells were transfected with HSP40 siRNA or Flag-HSP40, after 24 h normal incubation followed with 24 hr anaerobic incubation, RT-PCR was performed to analyze PDK1 mRNA level (mean ± S.D., n = 3).

HSPs family members are widely involved in regulating protein functions via posttranscriptional manners. Therefore the PKM2 protein stability and pyruvate kinase activity were analyzed in tumor cells with manipulating HSP40 expression. Over-expression or knockdown of HSP40 did not affect PKM2 mRNA level (Fig. S1B and S1C). However, PKM2 protein level was regulated by HSP40. Endogenous PKM2 protein level was apparently reduced while over-expressing HSP40 (Fig. 2A, 2B and 2C) but slightly accumulated in the absence of HSP40 (Fig. 2D and Fig. S1D). Previous studies showed that PKM2 was degraded by proteasome pathway under the regulation of the molecular chaperon HSC70 [27]. While HSP40 can bind to HSC70 and enhances the function of HSC70 dramatically [28], we wondered if the HSP40/PKM2 regulation was related to HSC70. As expected, HSP40 could immunoprecipitate HSC70 from cell lysates (Fig. 2E). In the presence of HSP40, HSC70-mediated degradation of PKM2 was enhanced (Fig. 2F). Consistent with protein level change, pyruvate kinase activity was significantly higher in HSP40 knockdown cells than that in control cells (Fig. 2G).

Besides, as a glycolytic enzyme in cytoplasm, PKM2 also regulates glucose metabolism as a transcriptional co-factor of Hif-1α in nucleus. Pyruvate dehydrogenase kinase 1 (PDK1) is one of downstream target gene of Hif-1α under-regulated by PKM2 co-activation. PDK1 is a switch of glycolysis and mitochondrial respiration, it phosphorylates and inhibits pyruvate dehydrogenase, an enzyme responsible for the conversion of pyruvate to acetyl-CoA, and further inhibits substrate to fuel the TCA cycle [29]. As shown in Fig. 2H and I, the mRNA level of PDK1 was down-regulated upon HSP40 over-expression and up-regulated upon HSP40 knockdown in hypoxia condition. Taken together, HSP40 can reduce the expression of endogenous PKM2 and at the same time impair its pyruvate kinase activity and co-factor function.

### HSP40-mediated glucose metabolic reprogramming was dependent on PKM2

**Figure 3:**
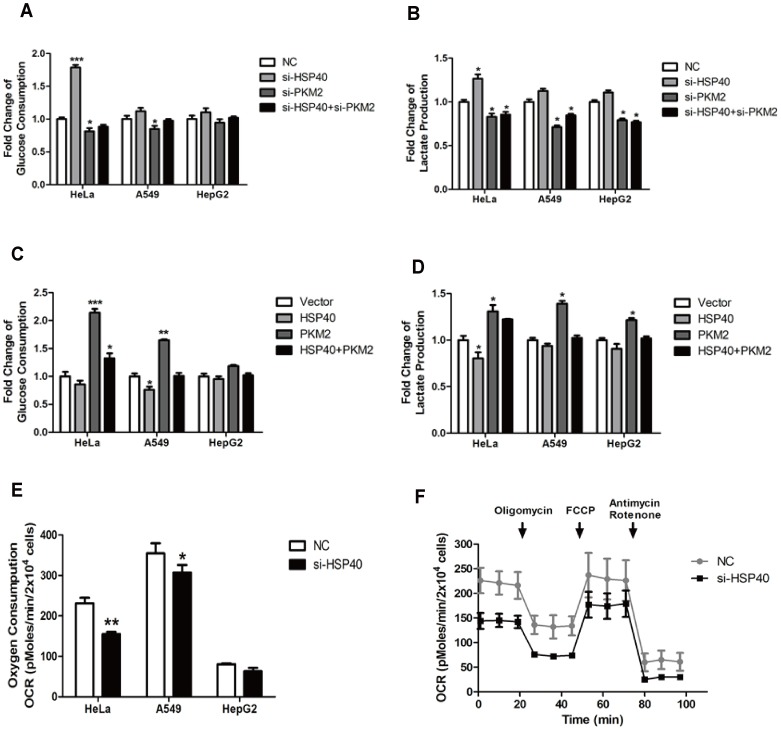
HSP40 regulates glycolysis and mitochondrial respiration in a PKM2 dependent way. (A and B) HeLa, A549 and HepG2 cells were transfected with the siRNAs of negative control (NC), PKM2 (si-PKM2), HSP40 (si-HSP40) or both. The media were collected for analysis of glucose consumption and lactate production (mean ± S.D., n = 3). (C and D) HeLa, A549 and HepG2 cells were transfected with empty vector, HA-PKM2 or both HA-PKM2 and Flag-HSP40. The media were collected to analysis of glucose consumption and lactate production (mean ± S.D., n = 3). (E) Basal OCR of HeLa, A549 and HepG2 cells after transfected with NC or si-HSP40 were detected by seahorse XF24 extracellular flux analyzer. (F) HeLa cells were transfected with NC or si-HSP40. 24 h after transfection, the cells were replanted to detect O_2_ consumption rate (OCR) by seahorse XF24 extracellular flux analyzer (mean ± S.D., n = 5). (mean ± S.D., n = 5).

PKM2 is considered as a key regulator of aerobic glycolysis in cancer cells. So we asked whether the HSP40-PKM2 interaction regulated cancer glucose metabolism. To investigate the role of HSP40 in glycolysis, the glucose consumption and lactate production were analyzed as indicators of glycolysis. Compared with negative control, both glucose consumption and lactate production were dramatically increased in cells treated with siRNA against HSP40. More importantly, this effect was dependent on PKM2 protein level. In the absence of PKM2, HSP40-mediated glycolysis was abolished (Fig. 3A and 3B). In addition, these findings were further supported by results of over-expressing experiments in cancer cells (Fig. 3C and 3D).

PKM2 functions as a switch of the glucose flux between glycolysis and TCA cycle. Therefore, we asked whether the mitochondrial oxygen respiration was also influenced by HSP40. Seahorse extracellular flux analysis combined of three pharmacological inhibitors, oligomycin, FCCP, Antimycin/Rotenon were used consecutively. As shown in Fig. 3E, the basal Cellular Oxygen Consumption Rate (OCR) indicated that HSP40 knockdown reduced oxygen consumption in cancer cells. Consistently, mitochondrial respiration was impaired while HSP40 was down-regulated (Fig. 3F). These data suggested that HSP40 shunted glucose from glycolysis to TCA cycle via PKM2.

### HSP40 impacted growth of tumor cells via PKM2

**Figure 4:**
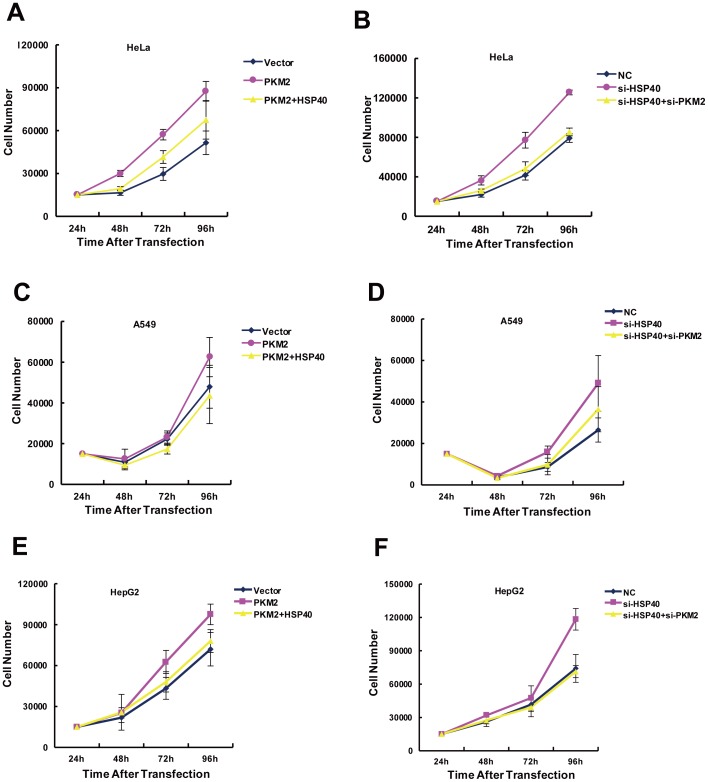
HSP40 impacts growth of HeLa cells via regulating PKM2 protein. (A, C and D) HeLa, A549 or HepG2 cells were transfected with HA-PKM2 or Flag-HSP40 or both. 24 h after transfection, cells were replanted and cell numbers were counted every 24 h for analysis of cell proliferation (mean ± S.D., n = 3). (B, D and E) HeLa, A549 or HepG2 cells were transfected with si-PKM2 or si-HSP40 or both. 24 h after transfection, cells were replanted and cell numbers were counted every 24 h for analysis of cell proliferation (mean ± S.D., n = 3).

Cancer cells reprogram their glucose metabolism, which is pivotal for cell proliferation. Since glucose consumption and lactate production were significantly changed by HSP40, we hypothesized HSP40 probably regulated proliferation of tumor cells. As expected, HeLa cells grew faster after knocking down of HSP40. However, HSP40 knockdown had no significant effect on cell growth in the absence of PKM2 (Fig. 4A). Furthermore, over-expressing HSP40 significantly inhibited cell growth, while PKM2 expression diminished the growth inhibition mediated by HSP40 knockdown (Fig. 4B). Besides HeLa cells, A549 cells (Fig. 4C and D) and HepG2 cells (Fig. 4E and F) also showed the same tendency. Taken together, HSP40 impaired the proliferation of tumor cells in a PKM2 dependent manner.

## Discussion

Many tumors exhibit significantly increased glucose utilization in comparison to normal tissues, namely Warburg effect [30]. The redirection of energy metabolism is largely orchestrated by proteins that are involved in programming the core hallmarks of cancer [31]. Growing evidences indicate that tumor suppressors and oncogenes reprogram glucose metabolism and impact tumor phenotypes via PKM2 [26]. Spoden et al. demonstrated that SUMO-E3 ligase interacts with PKM2. Their interaction promotes PKM2 entering the nucleus [32] and enhances Warburg effect [33]. In addition, structural studies reveal that the intermediate metabolites and small-molecule activators, such as FBP, serine, SAICAR, Vitamin K3, K5 and other synthetic PKM2 activators can bind to PKM2 and regulate its pyruvate kinase and protein kinase activity [24], [32]–[36]. In order to unveil more PKM2 regulation mechanisms, here we used yeast two-hydrid screening of human cDNA libraries to identify a new binding partner of PKM2. HSP40/DNAJB1 was found to interact with PKM2 (Fig.1). HSP40s are implicated in various human diseases, including cancers and neurodegenerative disorders [14], [37]. Heat shock proteins are over-expressed or totally lost in a wide range of human cancers. Recent research has revealed involvement of some of the DNAJ family members in various types of cancers [12].

As an isozyme specifically expresses in cancer cells, PKM2 plays an important role in the metabolism of cancer cells. Its functions could be mainly classified into three types according to previous studies, including pyruvate kinase activity, protein kinase activity and transcriptional co-factor function. In the present study, we found that knockdown of HSP40 increased the amount of PKM2 protein level and pyruvate kinase activity. Since PKM2 mRNA level had no significant changes, HSP40 binding may destabilize PKM2 protein via post-transcriptional mechanisms. The higher PKM2 enzyme activity was owing to PKM2 protein accumulation induced by HSP40 knockdown. Meanwhile, HSP40 association also regulated co-activator role of PKM2. The mRNA level of PDK1, one target gene of Hif-1α, was changed upon manipulating HSP40 expression (Fig. 2).

The present data suggested HSP40-PKM2 association had a comprehensive regulation on glucose metabolism, which was indicated by PKM2 own enzyme activity and PKM2-medidated PDK1 expression. As expected, HSP40 was involved in regulating glucose metabolism, which was dependent on PKM2 expression. HSP40 knockdown enhanced the glucose consumption and lactate production. However, HSP40 knockdown could not rescue the decrease of glycolysis caused by PKM2 knockdown. On the other hand, HSP40 inhibited PKM2 co-factor function. As mentioned above, PDK1 mRNA level was increased while HSP40 was down-regulated by siRNA. In the absence of PDK1 inhibition, PDH would shunt pyruvate into TCA cycle. As shown by oxygen consumption analysis, HSP40 promoted oxidative phosphorylation. Therefore, HSP40-PKM2 association was correlated with both pyruvate kinase itself and other glucose metabolic enzyme expression, which indicated their interaction was specifically involved in the Warburg effect (Fig. 3). In line with the inhibition effect of HSP40 on glycolysis and co-factor functions of PKM2, the cancer cell growth was also inhibited by HSP40 (Fig. 4). As reported earlier by Min Qi and colleagues, they found that HSP40/DNAJB1 inhibited cancer cell growth in vitro and in vivo by stabilizes MDM2 [21], which was consistent to our results although it worked though two different pathways.

Since our major conclusions are drawn from HSP40 knockdown data, it is important to eliminate potential off-target effects. So we synthesized another two well established HSP40 siRNAs according to published works [20], [21]. As we expected, the three siRNAs knockdown experiments shared the same conclusions (Fig. S1D–G).

**Figure 5:**
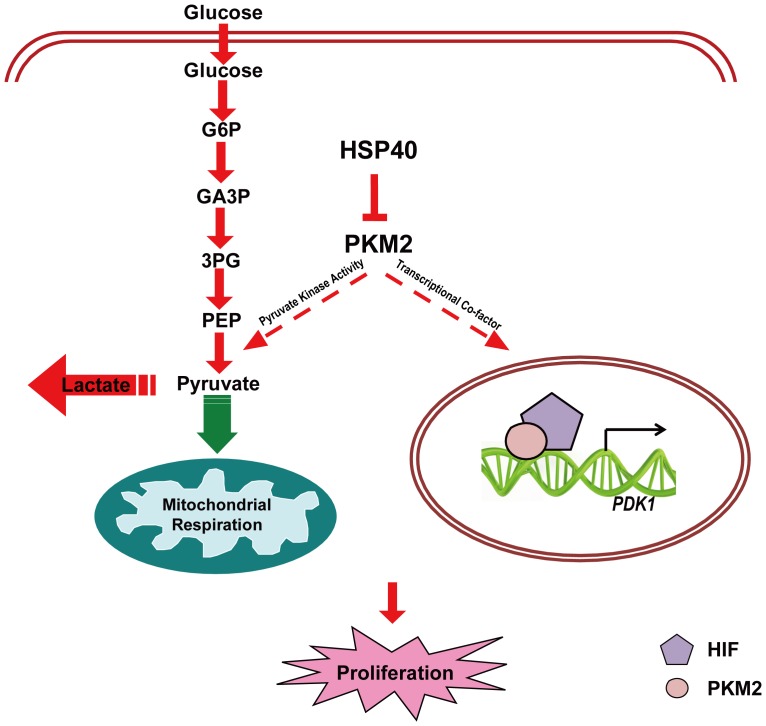
Cellular consequences followed by HSP40 binding to PKM2. HSP40 interacts with PKM2, inhibits PKM2 protein level and pyruvate kinase activity. Consequently, glucose uptake from medium decreases and lactate consumption drops as well and mitochondrial oxygen respiration rate ascends at the same time. Interestingly, the transcriptional co-factor function of PKM2 was also impacted. As a result of all these cellular PKM2 functions change, the proliferation of tumor cells was impacted to some degree. Red arrows and dashed lines indicated a decrease and green arrow indicated an increase.

In conclusion, HSP40 interacted with PKM2 to degrade it, and shunted glycolysis into oxidative phosphorylation. As a result, tumor cell proliferation was impacted by HSP40 in a PKM2 dependent manner (Fig. 5). Since PKM2 is a switch for glucose flux and a key regulator for cancer cell proliferation, it has been extensively studied for cancer therapy. Our study delineated HSP40-PKM2 association played an important role in PKM2-mediated glucose metabolic reprogramming and cancer cell growth. It suggested that targeting both HSP40 and PKM2 could be an effective therapeutic approach.

## Supporting Information

Figure S1 (A) The knockdown efficiency of PKM2 siRNA (si-PKM2) detected by Western Blotting. (B and C) The relative mRNA level of PKM2 in HeLa cells with HSP40 over-expressing or knockdown. (D) The knockdown efficiency and its impact on PKM2 protein levels of 2# and 3# HSP40 siRNA (si-HSP40) detected by Western Blotting. (E and F) HeLa cells were transfected with the siRNA of negative control (NC), PKM2 (si-PKM2), HSP40 (2# or 3# si-HSP40) or both. The media were collected for analysis of glucose consumption and lactate production (mean ± S.D., n = 3). (G) HeLa cells were transfected with si-PKM2 or si-HSP40 or both. 24 h after transfection, cells were replanted and cell numbers were counted every 24 h for analysis of cell proliferation (mean ± S.D., n = 3).
(TIF)
