# Supplementary figures and images for: HSP40 Interacts with Pyruvate Kinase M2 and Regulates Glycolysis and Cell Proliferation in Tumor Cells
Source: PLoS One. 2014 Mar 21;9(3):e92949. doi: 10.1371/journal.pone.0092949 (PMC3962495; doi:10.1371/journal.pone.0092949)

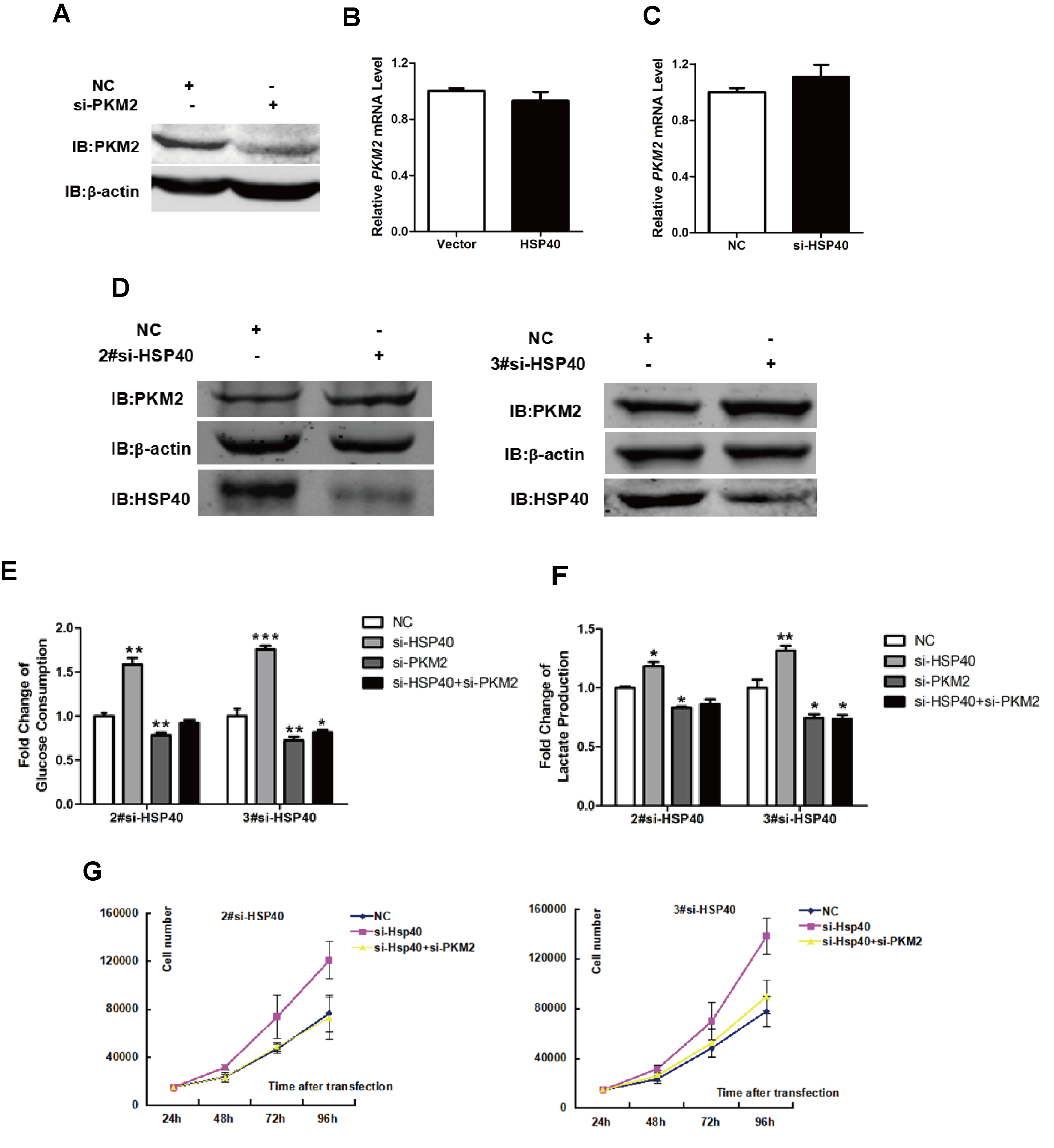

Supplement: Figure S1 — (A) The knockdown efficiency of PKM2 siRNA (si-PKM2) detected by Western Blotting. (B and C) The relative mRNA level of PKM2 in HeLa cells with HSP40 over-expressing or knockdown. (D) The knockdown efficiency and its impact on PKM2 protein levels of 2# and 3# HSP40 siRNA (si-HSP40) detected by Western Blotting. (E and F) HeLa cells were transfected with the siRNA of negative control (NC), PKM2 (si-PKM2), HSP40 (2# or 3# si-HSP40) or both. The media were collected for analysis of glucose consumption and lactate production (mean ± S.D., n = 3). (G) HeLa cells were transfected with si-PKM2 or si-HSP40 or both. 24 h after transfection, cells were replanted and cell numbers were counted every 24 h for analysis of cell proliferation (mean ± S.D., n = 3). (TIF) [file pone.0092949.s001.tif]
